# A High Fat Diet during Adolescence in Male Rats Negatively Programs Reproductive and Metabolic Function Which Is Partially Ameliorated by Exercise

**DOI:** 10.3389/fphys.2017.00807

**Published:** 2017-11-02

**Authors:** Carlos A. Ibáñez, Rafaela P. Erthal, Fernanda M. Ogo, Maria N. C. Peres, Henrique R. Vieira, Camila Conejo, Laize P. Tófolo, Flávio A. Francisco, Sandra da Silva Silveira, Ananda Malta, Audrei Pavanello, Isabela P. Martins, Paulo H. O. da Silva, Lucas Paulo Jacinto Saavedra, Gessica D. Gonçalves, Veridiana M. Moreira, Vander S. Alves, Claudinéia C. da Silva Franco, Carina Previate, Rodrigo M. Gomes, Renan de Oliveira Venci, Francielle R. S. Dias, James A. Armitage, Elena Zambrano, Paulo C. F. Mathias, Glaura S. A. Fernandes, Kesia Palma-Rigo

**Affiliations:** ^1^Reproductive Biology Department, Instituto Nacional de Ciencias Médicas y Nutrición Salvador Zubirán, Mexico City, Mexico; ^2^Laboratory of Secretion Cell Biology, Department of Biotechnology, Genetics and Cell Biology, Universidade Estadual de Maringá, Maringá, Brazil; ^3^Laboratory of Toxicology and Reproductive Metabolic Disorders, Department of General Biology, Universidade Estadual de Londrina, Londrina, Brazil; ^4^Laboratory of Endocrinology and Metabolism, Department of Physiological Sciences, Universidade Federal de Goiás, Goiânia, Brazil; ^5^School of Medicine, Deakin University, Waurn Ponds, VIC, Australia

**Keywords:** high fat diet, moderate exercise, glucose metabolism, obesity, reproductive system

## Abstract

An interaction between obesity, impaired glucose metabolism and sperm function in adults has been observed but it is not known whether exposure to a diet high in fat during the peri-pubertal period can have longstanding programmed effects on reproductive function and gonadal structure. This study examined metabolic and reproductive function in obese rats programmed by exposure to a high fat (HF) diet during adolescence. The effect of physical training (Ex) in ameliorating this phenotype was also assessed. Thirty-day-old male Wistar rats were fed a HF diet (35% lard w/w) for 30 days then subsequently fed a normal fat diet (NF) for a 40-day recovery period. Control animals were fed a NF diet throughout life. At 70 days of life, animals started a low frequency moderate exercise training that lasted 30 days. Control animals remained sedentary (Se). At 100 days of life, biometric, metabolic and reproductive parameters were evaluated. Animals exposed to HF diet showed greater body weight, glucose intolerance, increased fat tissue deposition, reduced VO_2max_ and reduced energy expenditure. Consumption of the HF diet led to an increase in the number of abnormal seminiferous tubule and a reduction in seminiferous epithelium height and seminiferous tubular diameter, which was reversed by moderate exercise. Compared with the NF-Se group, a high fat diet decreased the number of seminiferous tubules in stages VII-VIII and the NF-Ex group showed an increase in stages XI-XIII. HF-Se and NF-Ex animals showed a decreased number of spermatozoa in the cauda epididymis compared with animals from the NF-Se group. Animals exposed to both treatments (HF and Ex) were similar to all the other groups, thus these alterations induced by HF or Ex alone were partially prevented. Physical training reduced fat pad deposition and restored altered reproductive parameters. HF diet consumption during the peri-pubertal period induces long-term changes on metabolism and the reproductive system, but moderate and low frequency physical training is able to recover adipose tissue deposition and reproductive system alterations induced by high fat diet. This study highlights the importance of a balanced diet and continued physical activity during adolescence, with regard to metabolic and reproductive health.

## Introduction

Obesity is a global health problem, which is related to a sedentary lifestyle and highly calorific diets, which are rich in simple sugars and fat. In the last 40 years the rates of obesity in reproductive-age men has increased, with 36.9% of the population classified as overweight or obese in 2013 (Ng et al., 2014). Obesity underlies impaired glucose metabolism but may also decrease male reproductive potential (McPherson and Lane, 2015). This may further affect gamete quality and impact upon the health of the developing fetus and subsequent offspring (McPherson et al., 2014). Furthermore, the comorbidities and consequences of obesity impose a huge economic burden to public health management, including costs of cardiometabolic diseases and infertility treatments.

It is suggested that obesity negatively impacts sperm function, even if this function does not render obese males infertile. Studies have shown that almost 80% of men that present to fertility clinics are classified as either overweight or obese (Bakos et al., 2011). Furthermore, obesity modifies the reproductive system in conjunction with diabetes in obese men (Kriegel et al., 2009). Increased adiposity may not be the sole driver of impaired reproductive function in obese males, with comorbidities such as Type 2 Diabetes also influencing reproductive health (McPherson and Lane, 2015). However, little is known about the relationship between these alterations and the factors that modulate or determine reproductive health in obese individuals.

Previous studies provide evidence that adult obesity, metabolic dysfunction and reproductive impairment may be programmed by insults during early phases of life (Armitage et al., 2004; McMillen and Robinson, 2005; Zambrano et al., 2014). This process of programming adult disease by modulating the environment encountered during development is termed the developmental origins of health and disease (DOHaD). Maternal obesity or maternal protein restriction both lead to altered testicular morphology and elevated oxidative stress in the offspring (Rodriguez-Gonzalez et al., 2014, 2015). Recently, we have reported that adolescence is another window for programming of metabolic dysfunction (de Oliveira et al., 2013). Adequate nutrition during adolescence is very important due the rapid physical, psychosocial, sexual, and cognitive maturation processes occurring at this life stage (Salam and Bhutta, 2015). Furthermore, in humans dietary behavior during adolescence appears to contribute to obesity, including low meal frequency, skipping breakfast, and a high consumption of sugar sweetened beverages (Moreno et al., 2010). The impact of obesity on pubertal timing has been well addressed in the literature (Santos et al., 2015). However, no studies have addressed the effect of obesity programmed during adolescence on the morphology of adult testis and sperm. Data from our group shows that exposure to a high fat diet during adolescence programs the development of obesity and subsequent deregulation of glucose metabolism (Gomes et al., 2016); however, it is not known whether this is associated with any impact upon reproduction system structure or function.

Several studies have reported the benefit of physical exercise to metabolism and control of obesity (Henriksen, 2002; Levin, 2005; Gaesser et al., 2011; Zouhal et al., 2013). Exercise has been considered an efficient stimulus to control body weight gain and metabolic dysfunction in organisms programed to obesity (Andreazzi et al., 2009; Gomes et al., 2013). It has also been shown that impaired sperm quality and fertility potential in rat offspring from obese dams can be ameliorated by exercise performed during adulthood (Santos et al., 2015). We hypothesized that high fat diet exposure during adolescence induces long term dysfunction of the metabolic and reproductive systems but physical exercise ameliorates function. In this context, the present study aimed to investigate the effect of moderate physical training on reproductive system and metabolism of obese rats programed by high fat (HF) diet during adolescence.

## Materials and methods

### Experimental model

Twenty-five-day-old Wistar male rats were supplied by the State University of Maringa central animal facility and maintained in the animal facility of the Laboratory of Secretion Cell Biology. Animals were group housed (five rats per cage), under controlled conditions (temperature: 22 ± 2°C; photoperiod: 07 h 00 min—19 h 00 min), with water and food provided *ad libitum* throughout the experimental period. Experimental assays were performed on 10 animals per group from 40 different litters, where n represents the litter number.

**Figure 1:**
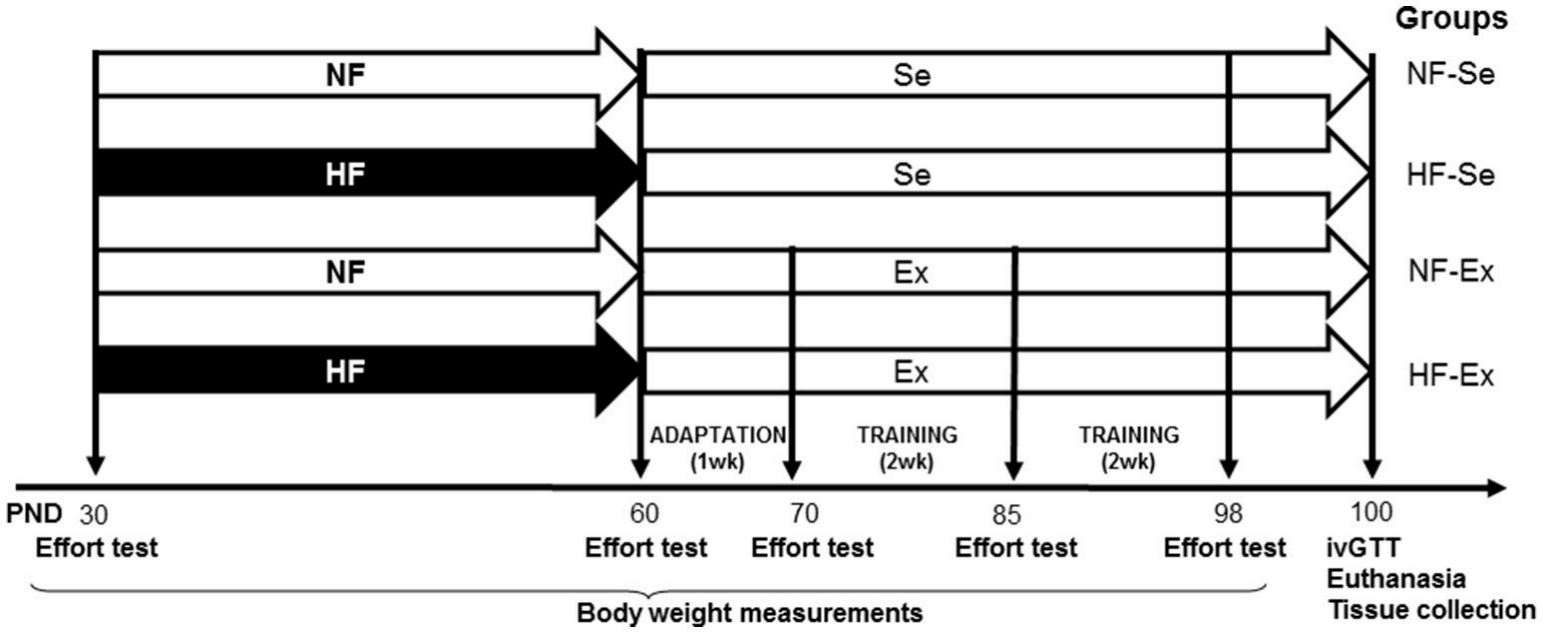
Experimental design. NF, normal fat diet animals; HF, high fat diet animals; Se, sedentary animals; Ex, exercised animals.

After 5 days of environmental adaptation, a group of 30-day-old animals were fed a high fat diet for 30 days (HF; 35% lard; 5.817 kcal/g) (Barella et al., 2012). From 60 days of life onwards they were fed with a commercial diet (NF; 3.801 Kcal/g; AIN 93 M, Nuvital®, Curitiba/ PR, Brazil). Control rats were fed with the commercial diet thought the experimental protocol (NF; 3.060 Kcal/g; AIN 93 M, Nuvital®, Curitiba/ PR, Brazil). At 60 days of life animals commenced a treadmill running adaptation protocol consisting of 5 sessions of low activity (defined as 55–65% of VO_2max_) and then at 70 days an exercise training protocol that lasted 30 days, creating 4 groups: (1) animals exposed to normal fat (NF) diet that remained sedentary (NF-Se); (2) animals exposed to the NF diet and exposed to exercise training (NF-Ex); (3) animals exposed to the HF diet that remained sedentary (HF-Se), and (4) animals exposed to the HF diet and exercise training (HF-Ex) (Figure 1). At 100 days of life experimental data were collected.

All animal procedures were conformed with the federal laws in Brazil for experimental uses of animals and approved by the Ethical Committee for Animal Experiments of the Universidade Estadual de Maringa.

### Moderate treadmill physical training protocol

Animals were trained, always in the morning, on a specialized treadmill for rodents (Panlab, Harvard Apparatus®, Cornellà- Barcelona—Spain). Electric shocks (0.2 e 0.4 mA) were used as a stimulus to keep the animal moving on the treadmill (Brooks and White, 1978; Radahmadi et al., 2013). Initially animals were adapted to running in the treadmill at low speed, starting at 16 cm/s for 12 min in the first session and finishing at 20 cm/s for 20 min in the last session (5 sessions from 60 to 69 days of life). The formal training period consisted of exercise performed three times a week for 4 weeks (12 sessions from 70 to 98 days of life). Each session consisted of 2 min of warm-up at 16 cm/s followed by 40 min of exercise of at moderate intensity and finally 2 min at 16 cm/s as a cool down period. Exercise of moderate intensity corresponded to a treadmill velocity that elicited between 55 and 65% of VO_2max_ and was adjusted to the value of final workload achieved in effort tests (average 50 cm/s). The intensity of the training was confirmed at 70, 85, and 98 days of life using a maximum effort test (Tofolo et al., 2015).

### Effort test

At 30, 60, 70, 85, and 98 days of life, physical trained (Ex) animals performed an effort test to determine VO_2max_ utilizing a gas analyzer coupled to a hermetically sealed cage with an individual treadmill for rodents (Panlab, Harvard Apparatus®, Cornellà- Barcelona—Spain). The test began with a warm up (5 min/10 cm/s/0° of inclination), followed by increments of 9 cm/s every 3 min until exhaustion of the animal; defined as when the animal was unable to keep the pace set by the treadmill (Ferreira et al., 2007; Tofolo et al., 2015). The final workload was considered to be the velocity at which animals were exhausted and were not able to maintain their running velocity. The VO_2max_ was the average of the last 30 s of O_2_ consumption achieved before the effort test finished. VO_2max_ and energy expenditure were determined by Metabolism software (version V.2.2.01 for Windows, Panlab, Harvard Apparatus®, Cornellà- Barcelona - Spain) in combination with a gas analyzer.

### Body weight gain

Body weight was monitored once a week between 30 and 98 days of life.

### Intravenous glucose tolerance test (ivGTT)

A silicone cannula was implanted into the right jugular vein of 99-day-old rats under anaesthesia (ketamine/xylazine, 0.5 mg/100 g of body weight). The following day, an ivGTT was performed as previously described(de Oliveira et al., 2011). Briefly, after 12-h fasting, intravenous glucose (1 g/kg of bodyweight) was injected into the cannula. Blood samples (0.3 mL) were collected prior to the glucose load (0 min) and then at 5, 15, 30, and 45 min. A corresponding volume of saline (0.9%) was intravenously injected to maintain volaemia. Plasma was used to determine glycaemia by the glucose-oxidase technique (Gold Analisa® Belo Horizonte/MG, Brazil). Increases in total glycaemia were calculated, after subtracting fasting values, using the areas under the glycaemia curves for the 45 min of ivGTT.

### Fat pad evaluation

Animals were killed (at 100 days of life) by decapitation. Fat pad stores (mesenteric, retroperitoneal and periepididymal) were removed and weighed to evaluate adiposity. The percentage of fat relative to the total animal body weight was used to estimate fat accumulation. Male gonads were removed (right testis and right epididymis) to assess evaluate reproductive system morphology. Liver and muscle (gastrocnemious and soleous) were also collected.

### Daily sperm production per testis, sperm number and transit time in the epididymis

Decapsulated right testis and epididymis were weighed and homogenized as described previously by Robb et al. (1978), with the adaptations described previously (Siervo et al., 2015). After dilution of homogenates, a small aliquot of sample was transferred to a Neubauer chamber (4 fields per animal) for counting homogenization- resistant spermatids (that corresponds to the stage 19 of the spermatogenesis) in the testis, and spermatozoa in epididymis. To calculate the daily sperm production (DSP), the concentration of homogenization-resistant spermatids per testis was divided by 6.1, which is the number of days during which mature spermatids remain in the seminiferous epithelium. To calculate sperm transit time through the epididymis, the number of sperm in each epididymal portion was divided by DSP.

### Sperm morphology

Contents of the vas deferens were removed by internal rinsing with 1.0 mL of 10% formal saline. Smears were prepared on histological slides from this solution and observed with an Opton photomicroscope (400x magnification). Two hundred spermatozoa were analyzed per animal. Morphological analyses were classified into three general categories: normal morphology, head abnormalities (without characteristic curvature or isolated form, i.e., no tail attached) and tail abnormalities (broken, rolled into a spiral and isolated, i.e., no head attached). This analysis was performed as described by Fernandes et al. (2007).

### Histological analyses of reproductive system

The left testis and epididymis (*n* = 5 five per group) were removed, fixed in Bouin Solution, embedded in Paraplast® and exhaustively sectioned at 5 μm. Testis and epididymis sections were stained with hematoxylin and eosin and examined for general histopathological and morphometric analysis as described by Favareto et al. (2011).

#### Seminiferous tubule diameters and seminiferous epithelium height

Ten random seminiferous tubules, per animal, in stage IX of the seminiferous epithelium cycle, were examined. Seminiferous tubule diameters were measured with an Opton photomicroscope (400x magnification) and BEL view software (version 6.2.3.0 for Windows). Likewise, the seminiferous epithelium height was measured in the same tubules using the methodology described above. In each seminiferous tubule, the mean of four measures for diameters and height was calculated and used in the statistical analysis.

#### Histopathological analysis in testis

Histological analyses were performed in 100 randomly selected testicular cross-sections per animal, using an Opton microscope (100x and 400x magnification). The seminiferous tubules were divided into normal or abnormal, according to the appearance of cells present in the seminiferous tubules. Abnormal tubules were subdivided into: immature germ cells in the lumen; acidophilic cells and the presence of vacuolization.

#### Spermatogenesis kinetics

One hundred random tubular sections per animal were classified into four categories: stages I–VI, VII–VIII, IX–XIII, and XIV of the seminiferous epithelium cycle, according to the procedure described by Leblond and Clermont (1952), under a light microscope (Opton) at 100x and 400x magnification.

#### Stereological and histopathology analysis in the epididymis

For stereological analysis of the epididymis, 10 random epididymal cross-sections per animal were selected and analyzed (Siervo et al., 2015). This analysis was performed by means of Weibel's multipurpose graticule, with 168 points to compare the relative proportion of the epididymal components (epithelium, stroma and lumen) across experimental groups (50 sections per group in caput-corpus and cauda region. Histopathological inspection was performed qualitatively in Weibel's multipurpose graticulate caput-corpus and cauda per animal. These tissues were evaluated using on Opton microscope (100X and 400X magnification).

### Statistical analysis

Data are expressed as mean ± SEM. GraphPad Prism version 6.01 for Windows (GraphPad Software, La Jolla, CA, USA) was used for statistical analyses and graph design for results of biometric parameters, ivGTT and effort test. Statistical analysis was performed by two-way analysis of variance (ANOVA) followed by Tukey's multiple comparisons test. Factors were partitioned into diet (NF-HF) and physical exercise training (Se/Ex). A p value of less than 0.05 was considered significant considering the main effects of diet (NF-HF), exercise (Se-Ex) and their interaction (diet vs. exercise).

The variance among the experimental groups was compared by the Bartlett's test for results of reproductive system. Statistical analyses were performed by Kruskal–Wallis with *post-hoc* Dunn test. Data were expressed on mean ± SEM, percentage or interval between quartiles. Differences were considered significant when *p* < 0.05. The statistical analyses were performed by GraphPad InStat (version 3.01).

## Results

### Biometric parameters

**Figure 2:**
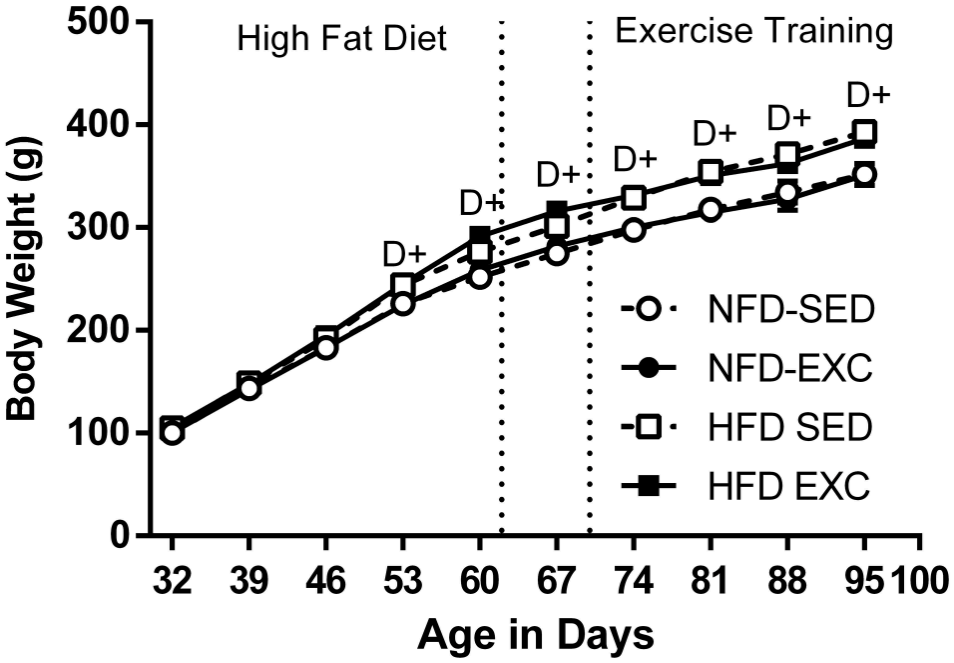
Body Weight evaluated thought experimental period (*n* = 10 per group). Values expressed as mean ± SEM. ^+^*P* < 0.05, for the probability based on analysis of variance. NF, normal fat diet animals; HF, high fat diet animals; Se, sedentary animals; Ex, exercised animals; D, effect of diet.

HF diet fed animals demonstrate greater body weight, compared with controls, from day 53 onwards (*p*_*D*_ < 0.05; Figure 2), corresponding to the second week of HF exposure.

Animals exposed to HF showed greater retroperitoneal, mesenteric and periepididymal fat deposition compared with NF rats (~ +40%, +35% and +24%, respectively; *p*_*D*_ < 0.01; Table 1). Physically trained animals demonstrated lower retroperitoneal fat deposition compared with sedentary animals (~-24%; *p*_*E*_ *P*_*E*_ < 0.05; Table 1).

**Table 1:** Biometric characteristics.

**Biometric parameters (g/100 g BW)**	**Se**		**Ex**		**D**	**E**	**I**
	**NF**	**HF**	**NF**	**HF**			
Retroperytoneal fat	1.13 ± 0.12^ab^	1.55 ± 0.14^ab^	0.80 ± 0.07^a^	1.25 ± 0.12^b^	+++	+	NS
Mesenteric fat	0.64 ± 0.07^a^	0.86 ± 0.05^b^	0.57 ± 0.04^a^	0.76 ± 0.02^ab^	+++	NS	NS
Periepididymal fat	1.07 ± 0.09^a^	1.39 ± 0.08^b^	1.12 ± 0.06^ab^	1.33 ± 0.10^ab^	++	NS	NS
Gastrocnemious muscle	0.52 ± 0.03	0.63 ± 0.03	0.53 ± 0.03	0.63 ± 0.05	++	NS	NS
Soleous muscle	0.046 ± 0.002	0.051 ± 0.003	0.048 ± 0.005	0.047 ± 0.003	NS	NS	NS
Liver	3.33 ± 0.13^a^	3.53 ± 0.04^ab^	3.35 ± 0.09^a^	3.71 ± 0.04^b^	++	NS	NS
Right testis	0.40 ± 0.01^a^	0.36 ± 0.009^b^	0.44 ± 0.01^a^	0.38 ± 0.01^ab^	+++	+	NS
Right epididymis	0.18 ± 0.02	0.16 ± 0.01	0.19 ± 0.02	0.17 ± 0.01	NS	NS	NS

*Values expressed as mean ± SEM. Different letters indicate groups that differ statistically (p < 0.05) based on Tukey's multiple comparisons test. + P < 0.05, +++ P < 0.001 for the probability based on analysis of variance. NF, normal fat diet animals; HF, high fat diet animals; Se, sedentary animals; Ex, exercised animals; D, effect of diet; E, effect of exercise; I, interaction between diet and exercise, NS, no significant difference*.

The mass of the gastrocnemius muscle was ~20% greater in HF animals compared with NF animals (*p*_*D*_ < 0.01; Table 1), but exercise did not affect this parameter. Soleus muscle weight was not affected by any factor.

Liver mass was approximately 8% greater in HF animals compared with NF animals (*p*_*D*_ < 0.01; Table 1).

Testicular mass was approximately 11.5% greater in HF animals compared with NF animals (*p*_*D*_ < 0.001; Table 1) and animals exposed to exercise demonstrated a 7% increase in testis weight compared with sedentary animals (*p*_*E*_ < 0.05; Table 1).

### Reproductive system

#### Sperm number, daily sperm production, sperm transit time through caput/corpus epididymal and spermatic morphology

Sperm count parameters both in testis and epididymis are shown on Table 2. The analyses of spermatic parameters showed that neither HF nor physical exercise were able to alter sperm number in the testis and DSP.

**Table 2:** Sperm counts.

	**Se**		**Ex**	
	**NF**	**HF**	**NF**	**HF**
**TESTIS**				
Sperm number (× 10^6^)	134.8 ± 8.3	114.3 ± 16.4	148.7 ± 13.7	135.1 ± 12.0
Sperm number per gram (× 10^6^)	119.7 ± 10.9	101.4 ± 7.5	115.2 ± 7.9	102.9 ± 8.7
Daily sperm production (× 10^6^)	22.1 ± 1.3	18.7 ± 2.7	24.4 ± 2.2	22.1 ± 1.9
**CAPUT AND CORPUS EPIDIDYMIS**				
Sperm number (× 10^6^)	78.4 ± 22.5	58.1 ± 10.5	73.4 ± 4.2	76.6 ± 7.3
Sperm number per gram (× 10^6^)	305.3 ± 82.5	219.4 ± 41.7	284.4 ± 17.8	272.2 ± 32.2
Transit time (days)	3.7 ± 1.0	3.1 ± 0.6	3.3 ± 0.5	3.3 ± 0.4
**CAUDA EPIDIDYMIS**				
Sperm number in (× 10^6^)	130.5 ± 2.6^a^	78.3 ± 15.0^b^	78.8 ± 14.3^b^	85.1 ± 3.1^a^^b^
Sperm number per gram (× 10^6^)	745.4 ± 20.6^a^	389.8 ± 62.8^a^	416.2 ± 95.7^a^	458.2 ± 31.4^a^
Transit time (days)	6.3 ± 0.3	3.9 ± 0.6	3.5 ± 0.7	3.4 ± 0.3

*Values expressed as mean ± SEM. Kruskal–Wallis, with post-hoc Dunn test*.

a, b *Different letters indicate groups that differ statistically (p < 0.05)*.

Sperm number of the epididymis and sperm transit time in the epididymal caput and corpus were unchanged. Conversely, animals exposed only to high fat diet (HF-Se) or physical exercises (NF-Ex) showed a decrease in the number of sperm and number of sperm per gram in the cauda epididymis compared with animals from NF-Se group (Table 2). The animals exposed to both treatments were similar to all groups.

**Figure 3:**
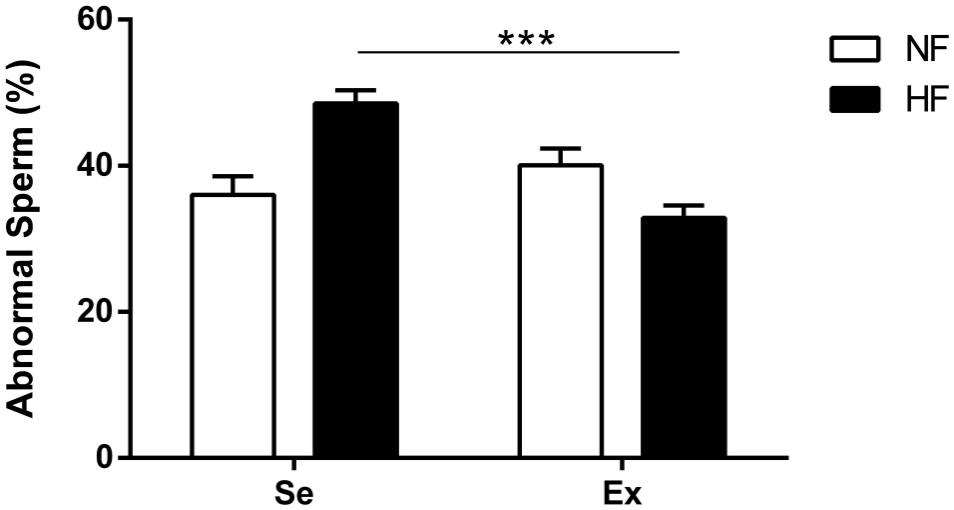
Sperm morphology in adult rats (*n* = 5 per group). Values expressed as percentage. ^***^p < 0.05, Kruskal–Wallis. with *post-hoc* Dunn test. NF, normal fat diet animals; HF, high fat diet animals; Se, sedentary animals; Ex, exercised animals.

In relation to spermatic morphology, HF-Ex animals showed reduced percentage of abnormal sperm compared with HF-SE (Figure 3).

#### Testicular morphometric and histopathological analyses

**Figure 4:**
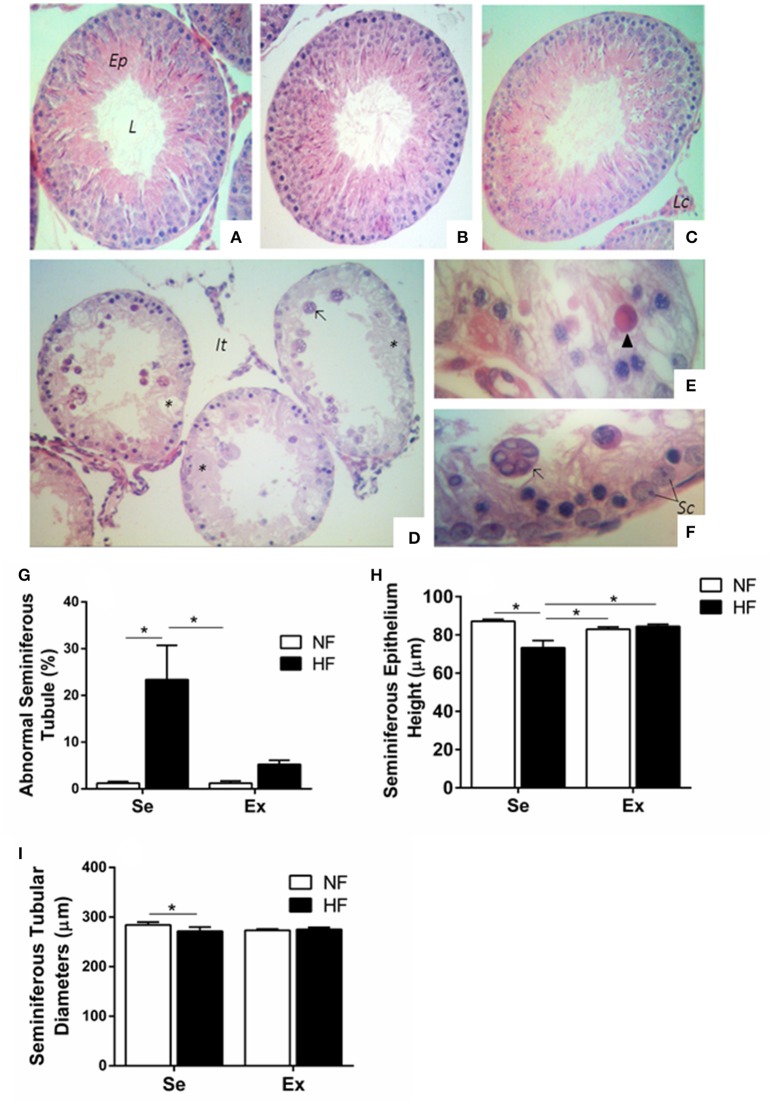
Histopathological analysis of seminiferous epithelium in testis in adult rats (*n* = 5 per group). Photomicrograph of testis sections from NF-Se **(A)**, NF-Ex **(B)**, HF-Ex **(C)**, and HF-Se **(D–F)** groups. **(A–C)** Observe the normal aspect of the seminiferous epithelium. **(D)** Note the presence of multinucleated cells in the lumen (arrow) and disruption of seminiferous epithelium (asterisk). **(E)** Acidophilic cell (head of arrow). **(F)** Shows greater magnification of multinucleated cell (arrow) of image **(D)**. **(G)** abnormal seminiferous tubules. **(H)** Seminiferous epithelium height. **(I)** Seminiferous tubular diameters. NF, normal fat diet animals; HF, high fat diet animal; Se, sedentary animals; Ex, Exercised animals; L, lumen; Ep, epithelium; It, interstitial tissue; Lc, Leydig cells; Sc, Sertoli cells. Hematoxylin and eosin stain. Magnification x100 **(A–D)**, x400 **(E,F)**. Values expressed as mean ± SEM. ^*^p < 0.05. Kruskal-Wallis test, with the *post-hoc* Dunn test. NF, normal fat diet animals; HF, high fat diet animals; Se, sedentary animals; Ex, exercised animals;

Figures 4A–D shows representative photomicrograph of testis sections from the groups. We observed that HF-Se group showed a reduction in seminiferous epithelium height and seminiferous tubular diameters, which was prevented by physical training (Figures 4H,I). It is important to point out that exercise coupled with a normal fat diet had no influence on any of these parameters. When compared to NF-Se, histopathological testicular analysis (Figure 4G) demonstrated that the exposure to a high fat diet led to an increase in the number of abnormal seminiferous tubules which presented acidophilic and multinucleated cells along with disruption of the seminiferous epithelium (Figures 4E,F). Exercise was associated with a reduction of this this damage in animals exposed to high fat diet in adolescence.

#### Spermatogenesis kinetics

Table 3 shows that exposure to a high fat diet decreased the number of seminiferous tubules in stages VII-VIII irrespective of exercise status (compared with the NF-Se group). On the other hand, exercise in NF fed animals had an opposite effect; the NF-Ex group showed an increase in stages XI-XIII when compared to NF-Se group. The proportion of tubules containing stage I to VI and XIV were statistically similar among all experimental groups.

**Table 3:** Spermatogenesis kinetics.

	**Se**		**Ex**	
	**NF**	**HF**	**NF**	**HF**
Stages I-VI	31.8 ± 1.02	35.6 ± 1.91	32.0 ± 1.48	37.0 ± 1.38
Stages VII-VIII	35.2 ± 1.07^a^	26.6 ± 0.98^b^	30.0 ± 2.02^a^^b^	28.3 ± 1.57^b^
Stages IX-XIII	28.4 ± 1.29^a^	32.2 ± 0.97^a^^b^	34.2 ± 0.97^b^	28.9 ± 0.61^a^
Stage XIV	4.6 ± 0.92	5.6 ± 1.21	3.8 ± 0.49	5.7 ± 0.58

*Values expressed as mean ± SEM. Kruskal–Wallis, with post-hoc Dunn test*.

a, b *Different letters indicate groups that differ statistically (p < 0.05)*.

#### Epididymal stereological analysis and histopathological analysis

Animals from the NF-Ex group showed an increase in the proportion of stromal compartment of epididymal caput in comparison with those from the NF-Se group (Table 4), however, animals exposed to the high fat diet did not differ statistically from NF-Se animals. Likewise, the proportion of stromal compartment in animals fed high fat diet did not differ statistically from NF-Ex group. In relation to epididymal cauda, the group HF-Ex presented a major proportion in luminal compartment and a reduction in the proportion of epithelium from animals of the same group in relation to NF-Se group. Histopathological analysis failed to indicate any variability between experimental groups.

**Table 4:** Stereological analysis of caput and cauda epididymis.

	**Se**		**Ex**	
	**NF**	**HF**	**NF**	**HF**
**CAPUT (2A)**				
Stroma	24.1 [21.4–28.0]^a^	23.2 [21.1–28.4]^a^	27.4 [23.9–32.4]^b^	28.0 [19.8–31.2]^a^^b^
Lumen	47.0 [43.4–50.0]^a^	48.2 [41.7–51.2]^a^	45.2 [40.8–50.0]^a^	49.4 [35.9–54.2]^a^
Epithelium	28.9 [25.1–32.4]^a^	28.0 [24.5–30.6]^a^	25.6 [23.8–29.6]^a^	25.6 [20.1–34.8]^a^
**CAUDA (5A/B)**				
Stroma	22.9 [18.7–28.0]^a^	22.6 [19.3–26.8]^a^	25.9 [21.4–30.3]^a^	22.0 [18.6–26.8]^a^
Lumen	52.4 [44.5–58.9]^a^^b^	50.6 [44.2–54.0]^a^	46.7 [43.4–52.5]^a^	54.2 [49.4–64.0]^b^
Epithelium	25.6 [23.1–29.3]^a^	27.7 [23.9–30.3]^a^	26.8 [22.2–30.9]^a^	21.1 [12.0–26.3]^b^

*Values expressed as median [Q1–Q3] from percentage. Kruskal–Wallis. with post-hoc Dunn test*.

a.b *Different letters indicate groups that differ statistically (p < 0.05)*.

### Metabolism

#### Intravenous glucose tolerance test (ivGTT)

**Figure 5:**
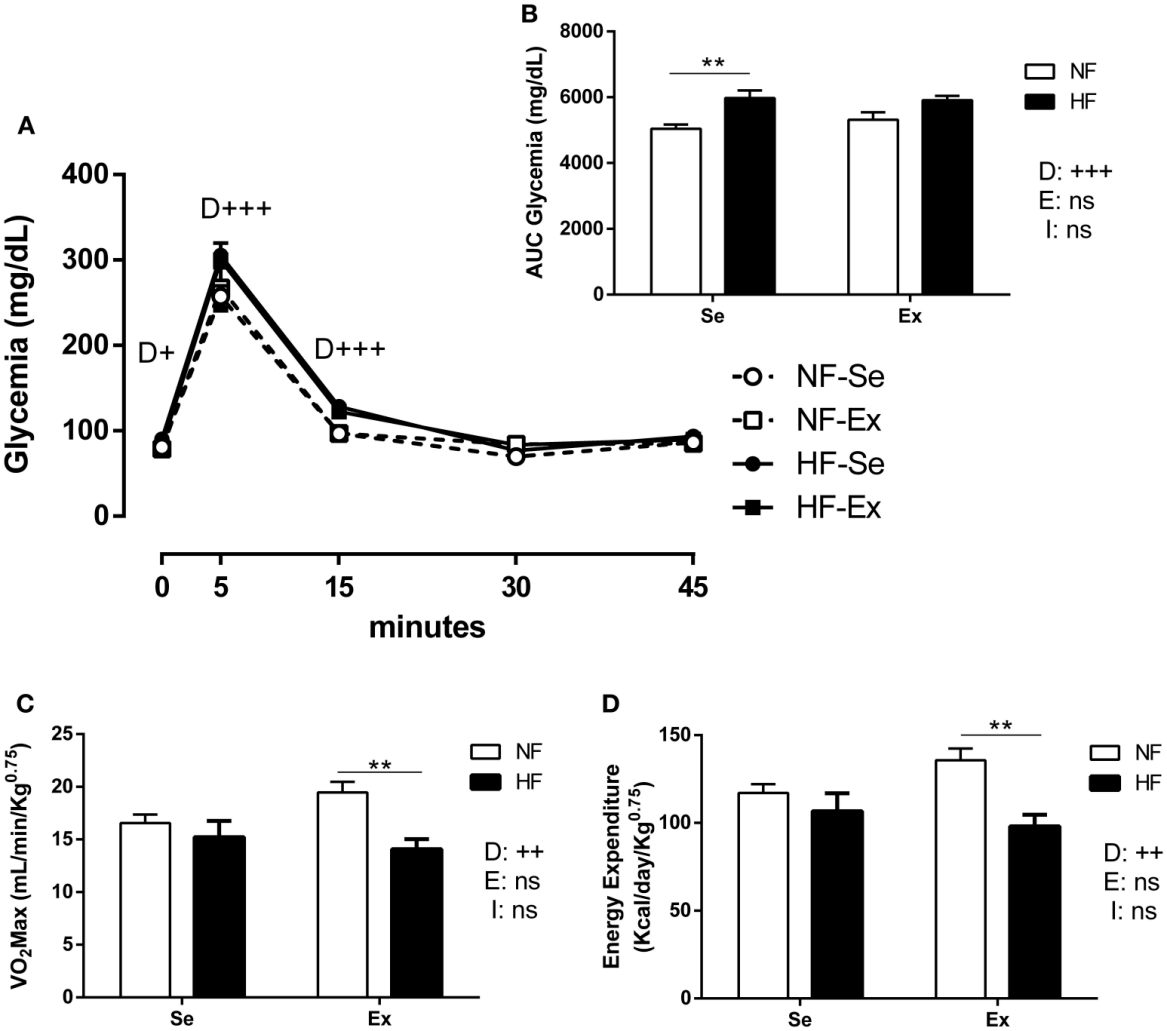
Glycaemia **(A)** and area under the curve (AUC) **(B)** during ivGtt. **(C)** VO_2max_, and **(D)** Energy expenditure in 98 day-old rats (*n* = 10 per group) Values expressed as mean ± SEM. ^**^p < 0.01, of the differences between HF and NF rats for the probability based on Tukey's multiple comparisons test. ^+^*P* < 0.05, ^++^*P* < 0.01, ^+++^*P* < 0.001 for the probability based on analysis of variance. NF, normal fat diet animals; HF, high fat diet animal; Se, sedentary animals; Ex, exercised animals; D, effect of diet; E, effect of exercise; I, interaction between diet and exercise; ns: no significant difference.

During the ivGTT, a peak in glycaemia was observed 5 min after glucose injection, and euglycaemia was restored within approximately 35 min (Figure 5A). At the peak of the curves, HF rats exhibited approximately 20% greater blood glucose concentration compared with NF animals, which translated to a greater glycaemia area under the curve (*p*_*D*_ < 0.001; Figures 5A,B). Exercise did not affect the glycaemia in either NF and HF groups.

#### Effort test

Two way ANOVA test shows a main effect of diet as animals exposed to the HF diet have reduced VO_2max_ and energy expenditure compared with NF animals (VO_2max_ and energy expenditure in Se: –9% and Ex: –27%, *p*_*D*_ < 0.01, Figures 5C,D). It was not observed a main effect of exercise in the VO_2max_ and energy expendititure (*p*_*E*_ = 0.44).

## Discussion

The major finding of the present study is that exposure to a high fat diet during adolescence lead to long-term changes in the reproductive system and metabolism of male rats, which may implicate reproductive and metabolic programming mechanisms (Metges, 2009; de Oliveira et al., 2013; Zambrano et al., 2014). Furthermore, a moderate intensity and low frequency moderate exercise training markedly improved reproduction markers in HF animals, by a mechanism largely independent of metabolic improvement. The present physical training protocol; low intensity, low frequency and short duration (30 days), may be too gentle to improve metabolism. However, the reproductive system appears to be more amenable to improvement at this level of physical activity. A more intense or more frequent physical exercise protocol (Santos et al., 2015) have been shown to induce both metabolic and reproductive rescue. It has been shown that the present protocol of physical exercise is able to ameliorate metabolic changes induced by a HF, when performed before the exposure to the dietary insult (Tofolo et al., 2015). Furthermore, physical training started earlier in life and performed until adulthood, concomitant with HF diet, promoted improvement of glucose and lipid homeostasis in adult animals compared with animals exposed to HF diet in the same conditions (Gomes et al., 2013).

Traditionally, there has been a great emphasis on the role of the fetal period in programming adult disease but fetal interventions are limited for obvious reasons. Previously, our group have identified other periods in life in which animals are susceptible to programming of diseases in adulthood (de Oliveira et al., 2013; Malta et al., 2015). Data from our group points that adolescence represents a window of susceptibility to programming. Exposure to a low protein or HF diet during adolescence programs cardiometabolic dysfunctions later in life (de Oliveira et al., 2013; Gomes et al., 2016). This, however is the first study to demonstrate the possibility of rescuing long term alterations in male reproductive system function caused by a dietary insult during adolescence with physical training. It is important to consider that adolescence is a period of intense neuroendocrine and reproductive changes, characterized by puberty and sexual development (Ebling, 2005; Salam and Bhutta, 2015), which contextualize the sensitivity of this phase of life and the present findings. It is well know that weight gain velocity increases and calories demand increases in the peri-pubertal period, which appear to affect puberty development. Indeed, obese adolescents demonstrate early puberty onset (Soliman et al., 2014). Furthermore, studies demonstrate that dietary patterns during adolescence have changed over the last 2–3 decades and affect body weight gain, which may contribute to overweight and obesity from this period of life onwards (Moreno et al., 2010).

The present study shows that consumption of HF induced significant alteration to testicular morphometric and histopathological analyses. A reduction in seminiferous epithelium height and seminiferous tubular diameter was observed in rats fed a high fat diet during adolescence only. Corroborating the data from our study, Erdemir et al. also showed a decrease in the number of normal seminiferous tubules of adult rats exposed to high fat diet for 10 weeks (Erdemir et al., 2012). In addition, a study by Liu et al. showed an increase in abnormal testicular structures, such as epithelial disruption, in 6 week old fat (20% fat) fed Sprague-Dawley rats (Liu et al., 2014). Conversely, we have shown previously that male Sprague-Dawley rats fed with HF (35% fat) from 21 until 90 days of life did not demonstrate abnormal seminiferous tubule morphology (Vigueras-Villasenor et al., 2011) suggesting that the dietary insult can affect the response to fat intake and exercise in a different way depending on the diet relative content of fat. A possible reason for these alterations is showed by Cano et al. (2008), whose study presented a reduction in levels of plasma testosterone after the male rats were exposed to HFD (35% fat) during 68 days of treatment (Cano et al., 2008) which was similar to the present study. Knowing the relevance of testosterone levels, especially during puberty enabling male sexual development and spermatogenesis (Johnson et al., 2015), a reduction on these levels could explain a possible mechanism for altering previous parameters.

The finding that exercise is protective is also dependent on a number of factors, including the age at which the intervention is introduced. Adult male rats exposed to swimming exercise for 4 weeks showed significant reduction of seminiferous tubule diameter in relation to control group, contrasting with our findings (Manna et al., 2004). The swimming exercise applied by Manna et al. (2004) is more intense than the one used in the present study. Furthermore, the same authors reported a decrease in spermatogenic cells at different stages of spermatogenesis, which may suggest an alteration in spermatogenesis kinetics as per the observation in the present. Conversely, a model using adult mice exposed to running training for 14 months showed positive effects on mice testicular health with regard to improved complete number and type of cells in seminiferous tubule, increased sperm density and more abundant Sertoli cells compared with that observed in sedentary mice (Chigurupati et al., 2008). Our findings demonstrate that a low frequency, low intensity physical exercise ameliorates the impairment caused by HF exposure. Over again, alterations in testosterone levels can impair the normal seminiferous tubule development. Study of Palmer et al. showed that exercise in mouse fed a high-fat diet restore levels of testosterone (Palmer et al., 2012).

The present study showed that testicular function was not altered in relation to the daily sperm production, but sperm morphology after treatment, was altered. Consistent with our data, Mortazavi et al. also failed to observe differences in sperm count between rats (3 months old) fed with high fat diet (444 kcal/100 g) and normal diet (304 kcal/100 grams) for 12 weeks (Mortazavi et al., 2014). The same author demonstrated other spermatic parameters were affected by the high fat diet such as an observation of reduced viability, motility and normal spermatic morphology. A study in adult mice (Palmer et al., 2012) demonstrates that a high fat diet (21% kcal as fat for 10 weeks) reduced sperm motility and morphology and DNA damage but a forced swimming exercise intervention resulted in normalized sperm motility and morphology, besides reduces the sperm DNA damage of rats (8 weeks old). The same study showed the differences in testosterone levels, which can be related with our results showing a concomitant normalization of plasma testosterone levels and sperm morphology. The Palmer study reported normal sperm count in fat fed animals; a finding that is consistent with our current observation. Our results shows that, both the high fat diet and exercise, whether isolated or in combination, can impair the proportion of the stage of spermatogenesis, which can be deleterious to spermatogenesis in the long run.

It has previously been suggested that consumption of a high fat diet (40% of calories as fat for 12 weeks) results in decreased antioxidant enzyme expression and function, thereby leading to cellular dysfunction. Moreover, antioxidant capacity is normalized in fat fed animals that are also submitted to endurance exercise training protocols (Alhashem et al., 2014). Other studies show that animals fed a high fat diet demonstrated alterations in spermatic parameters associated with oxidative stress, including reduced sperm concentration, viability, motility and DNA integrity (Vigueras-Villasenor et al., 2011; Chen et al., 2013; Mortazavi et al., 2014). In another study, increased adiposity as well as decreased sperm quality and fertility was shown in old (450 day) male Wistar rat offspring born to obese mothers. Importantly, 4 months of regular voluntary physical activity (from 330 days of life onwards), lead to lower adiposity index and an improved sperm quality and fertility. These beneficial effects were associated to decreased testicular oxidative stress biomarkers and increased sperm antioxidant activity found in exercised animals (Santos et al., 2015).

Epididymal tissue morphology demonstrated that the association between high fat diet and physical exercise impaired the normal development of both luminal and epithelial compartments of the epididymal cauda. Previous study have shown that epididymal regions respond in different ways after the same stimulus, with changes in some compartments of the epididymis caput and in others of the epididymis cauda induced by ethanol exposure during peripuberbal period (Paula Franco Punhagui et al., 2016). Despite of the alterations described, the organ function was not impaired since the spermatic transit time was unaltered within the caput / corpus or cauda epididymis. Our results corroborate with the study by Fernandez et al. which showed that sperm numbers in the caput/corpus was similar between animals fed a HFD for 15 weeks and controls (Fernandez et al., 2011).

There is no consensus statement regarding any beneficial or deleterious effects of physical exercise/sport on reproductive performance (Du Pressis et al., 2011). In fact, exercise has been shown to induce diverse effects on the reproductive system which vary with exercise type, intensity, time, order or characteristics of the physical exercise. For example, prolonged exhaustive exercise in men may lead to adverse effects on fertility, characterized by abnormal sperm morphology (Arce et al., 1993; Vaamonde et al., 2009). Interestingly, individuals who started extensive physical training at or around the time of puberty may be more susceptible to reproductive dysfunction (Vaamonde et al., 2016). In the present study, animals performed moderate and low frequency exercise for a short period, which was designed to avoid any adverse effects of exhaustive exercise on fertility. Therefore, the positive effect on reproductive function indicates that moderate exercise is a potential non-pharmacological treatment to improve reproductive function in obese individuals.

The present study also shows that animals exposed to HF and exercised showed an attenuation of fat deposits (mesenteric and retroperitoneal fats) which can be associated with protection of reproductive system which may suggest that adiposity levels are involved in the impairment of sperm function. Alternatively, Palmer et al. have shown that low intensity swimming training in mice improves reproductive system without affecting adiposity in obese animals, which suggest that adiposity itself is not the sole determinant in the impaired sperm function (Palmer et al., 2012). The different findings may be species or exercise dependent. Moreover, it may not be adiposity *per se* but rather glucose tolerance, which predicts sperm function. Blood glucose concentrations do correlate with the percentage of capacitated or non-capacitated sperm (Palmer et al., 2012). Accordingly, our present data show that animals exposed to HF during adolescence demonstrate obesity which is associated to glucose intolerance and dysfunction of reproductive system. Both hyperinsulinemia and hyperglycemia may underlie of altered sperm function, which occurs independent of adiposity (McPherson and Lane, 2015). Discordance between metabolism and adiposity in determining of reproductive dysfunction was also observed in the present study, as the moderate exercise improved adiposity index and sperm function without affecting glucose tolerance.

Very few studies have addressed adolescence as a period of plasticity in terms of developmental programming but the present study provides clear evidence that consumption of high fat diet during this period can have long-term effects on reproductive function. To conclude, HF exposure during adolescence induces to long term obesity, altered glucose metabolism and causes adverse effects on the male reproductive system, which may contribute to reproductive and metabolic programming. Furthermore, low frequency moderate exercise improves the reproductive deficit induced by this HF, which points to the importance of exercise as a potential non-pharmacological intervention to treat infertility in obese individuals programmed since adolescence.

## Author contributions

CI and RE contributed to the design of the study, acquisition, analysis and interpretation of data; revised critically the draft for important intellectual content; give final approval of the version to be published; and agree to be accountable for all aspects of the work. MP, HV, CC, LT, FF, FD, SS, AM, AP, IM, PS, LJ, FO, GG, VM, CF, VA, CP, RG, RV contributed with the acquisition; revised critically the draft for important intellectual content; give final approval of the version to be published; and agree to be accountable for all aspects of the work. JA and EZ participated in the interpretation of the data for the work; revised critically the draft for important intellectual content; give final approval of the version to be published; and agree to be accountable for all aspects of the work. PM, GF and KP conceived the study and participated in its design and coordination; drafted the manuscript; give final approval of the version to be published; and agree to be accountable for all aspects of the work.

### Conflict of interest statement

The authors declare that the research was conducted in the absence of any commercial or financial relationships that could be construed as a potential conflict of interest.

## Abbreviations

HF: high fat diet
NF: Normal fat diet
Ex: exercised animals
Se: sedentary animals
VO_2_max: maximal oxygen consumption during exercise
DOHaD: developmental origins of health and disease
IvGTT: Intravenous glucose tolerance test
DSP: daily sperm production.
